# Comprehensive Characterization for Ginsenosides Biosynthesis in Ginseng Root by Integration Analysis of Chemical and Transcriptome

**DOI:** 10.3390/molecules22060889

**Published:** 2017-05-31

**Authors:** Jing-Jing Zhang, He Su, Lei Zhang, Bao-Sheng Liao, Shui-Ming Xiao, Lin-Lin Dong, Zhi-Gang Hu, Ping Wang, Xi-Wen Li, Zhi-Hai Huang, Zhi-Ming Gao, Lian-Juan Zhang, Liang Shen, Rui-Yang Cheng, Jiang Xu, Shi-Lin Chen

**Affiliations:** 1 College of Pharmacy, Hubei University of Chinese Medicine, Wuhan 430065, China; zhangjingjing851@126.com (J.-J.Z.); zghu0608@163.com (Z.-G.H.); pwang54@yahoo.com.cn (P.W.); 2 Institute of Chinese Materia Medica, China Academy of Chinese Medicinal Sciences, Beijing 100700, China; suhe@cau.edu.cn (H.S.); swjs082lbs@126.com (B.-S.L.); smxiao@icmm.ac.cn (S.-M.X.); lldong@icmm.ac.cn (L.-L.D.); xwli@icmm.ac.cn (X.-W.L.); zlj0970@163.com (L.-J.Z.); lshen@icmm.ac.cn (L.S.); 18612545661@163.com (R.-Y.C.); 3 Guangdong Provincial Hospital of Chinese Medicine, The Second Affiliated Hospital of Guangzhou University of Chinese Medicine, and China Academy of Chinese Medical Sciences Guangdong Branch, China Academy of Chinese Medical Sciences, Guangzhou 510006, China; zhhuang7308@163.com; 4 Data Center, China Academy of Chinese Medicinal Sciences, Beijing 100700, China; leizhang@ndctcm.cn; 5 The Engineering Technology Research Center for Chinese Medicine, Henan Agricultural University, Zhengzhou 450002, China; gaozhiming672@sohu.com

**Keywords:** *Panax ginseng*, ginsenoside, transcriptome, triterpenes, biosynthesis

## Abstract

Herbgenomics provides a global platform to explore the genetics and biology of herbs on the genome level. *Panax ginseng* C.A. Meyer is an important medicinal plant with numerous pharmaceutical effects. Previous reports mainly discussed the transcriptome of ginseng at the organ level. However, based on mass spectrometry imaging analyses, the ginsenosides varied among different tissues. In this work, ginseng root was separated into three tissues—periderm, cortex and stele—each for five duplicates. The chemical analysis and transcriptome analysis were conducted simultaneously. Gene-encoding enzymes involved in ginsenosides biosynthesis and modification were studied based on gene and molecule data. Eight widely-used ginsenosides were distributed unevenly in ginseng roots. A total of 182,881 unigenes were assembled with an N50 contig size of 1374 bp. About 21,000 of these unigenes were positively correlated with the content of ginsenosides. Additionally, we identified 192 transcripts encoding enzymes involved in two triterpenoid biosynthesis pathways and 290 transcripts encoding UDP-glycosyltransferases (UGTs). Of these UGTs, 195 UGTs (67.2%) were more highly expressed in the periderm, and that seven UGTs and one UGT were specifically expressed in the periderm and stele, respectively. This genetic resource will help to improve the interpretation on complex mechanisms of ginsenosides biosynthesis, accumulation, and transportation.

## 1. Introduction

*Panax ginseng* C.A. Meyer, one of the world’s most important medicinal plants, has been commonly used as an adaptogenic or restorative drug in East Asia attributed to its many varieties of saponin and polysaccharide, and with numerous pharmaceutical effects related to the immunomodulation [1,2,3,4]. However, in recent years the naturally-occurring ginseng resources are being exhausted, along with their shrinking habitats [5]. Being a perennial understory herb, ginseng growth requires a particular environment, together with the effect of continuous cropping obstacles, there are fewer land resources suitable for ginseng cultivation: these factors seriously hinder ginseng production [6,7]. Ginsenosides are considered as the major bioactive compounds in *P. ginseng*. Until now, more than 150 ginsenosides have been identified, including Rg1, Rb1, Rc, Rd, Re, Rf, and so on [8,9,10]. It is extraordinarily difficult to synthesize ginsenosides artificially because of their complex structure. In view of this, synthetic biology techniques, such as tissue culture and bioconversion, are attractive strategies to produce ginsenosides and to solve the resource scarcity of *P. ginseng* [11,12,13]. Therefore, elucidating the biosynthetic pathways and regulatory mechanisms of ginsenosides will provide a foundation for the use of synthetic biology techniques.

Ginsenosides exhibited various among different species [14,15], varieties/cultivars [16], tissues [17,18], cultivation ages [19,20,21,22,23], and environmental conditions [24,25]. In addition to whole organs, some studies have focused on the accumulation and distribution patterns histologically of ginsenosides in different parts of the main root, which is considered as the major medicinal part [18,26,27,28,29]. In 1979, researchers reported that ginsenosides were mainly localized outside of the cambium in the main root by histochemical methods, and peeling as part of processing would dramatically reduce ginsenoside content [27]. Shu et al. established the matrix-assisted laser desorption/ionization mass spectrometry (MALDI-MS) method for the detection of unevenly distributed ginsenosides in the periderm and other inner portions of the main root [29]. Combined mass spectrometry imaging and other analytical methods, Liang and Bai also found that most ginsenosides were mainly localized in the periderm [26,28]. Specifically, some researchers have showed that multiple genes involved in the synthesis of ginsenosides are expressed in the phloem and xylem, which belong to the vascular system [30,31]. Namely, ginsenoside synthesis occurred in vascular tissue, such as the phloem, and are then transported to the root periderm and thin wall cells with the transporters playing a defensive role against animals and insects [17,32,33,34]. Hence, gene information in different parts of the main root is vital to find the ginsenoside biosynthesis genes, elucidate the biosynthetic pathways and regulatory mechanisms.

To date, for ginseng, very large amounts of transcriptome data has been reported on the basis of its hairy root [35], different organs [36,37], and different year-old roots [37,38]. The transcriptome information greatly accelerates the finding of functional genes involved in ginsenoside biosynthesis, and it has been applied to improve the interpretation for analyzing of metabolic regulation, molecular assisted breeding, and the screening of molecular markers and resistance genes in the absence of genomic information [36,38,39,40]. However, previous reports mainly discussed the transcriptome of ginseng at the organ level, and there are no studies that report on the transcriptome of the main root by further subdivision histologically. Here, ginseng roots were separated into three tissues (periderm, cortex, and stele), where each tissues had five duplicates. The chemical analysis and transcriptome analysis were conducted simultaneously. Analysis combining the metabolome and transcriptome provides new insights on the comprehensive characterization for ginsenoside biosynthesis in ginseng root, and serves as a valuable resource for further study on the biological mechanisms of ginsenoside synthesis, accumulation, and transportation.

## 2. Results

### 2.1. Localization of Ginsenosides in the Main Root of P. ginseng

**Figure 1:**
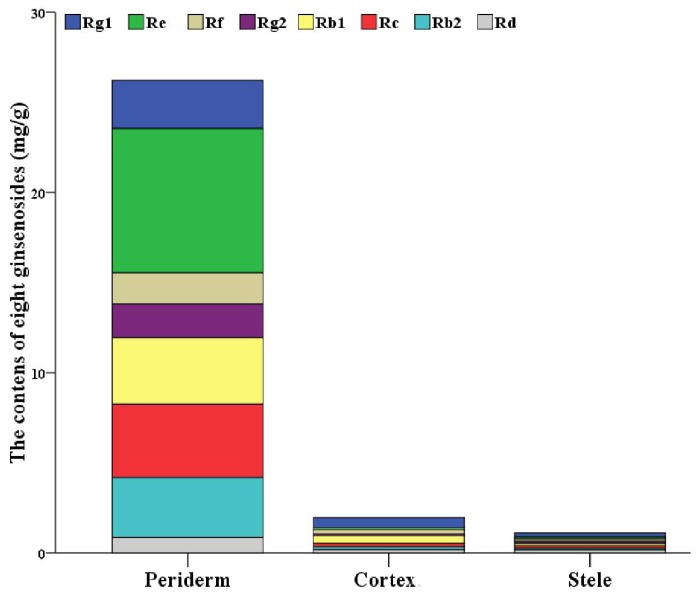
Bar graph for the distribution of eight ginsenosides in ginseng main root tissues.

The results showed that the eight ginsenosides varied in the three tissues (Supplementary Materials Figure S1). All eight ginsenosides’ average contents in the periderm were higher than those in the other two tissues, ranging from 4.6-fold to 74-fold. The content of individual ginsenoside-Re was the highest in the periderm as 8.001 mg/g. The content of individual ginsenoside-Rg1 was the highest in both cortex and stele, with 0.552 mg/g and 0.228 mg/g, respectively. However, the content of eight ginsenosides in the cortex was comparable to that in the stele, but significantly lower than that in the periderm (Figure 1, Table S1). All eight identified ginsenosides were found in the periderm, while ginsenoside-Rg2 was not detected in some of cortex samples and stele samples. Comparison of the five replicates showed a great variation in the contents of ginsenosides in different individuals. In the periderm of individual-3, the contents of the majority ginsenosides reached the highest, while individual-2 had the lowest accumulation. Considering the cortex and stele of individual-5, the contents of the majority ginsenosides reached the highest levels, while individual-1 had the lowest accumulation (Table S1). This confirms that ginsenosides are unevenly distributed in ginseng root, and it demonstrates that ginsenosides are mainly accumulated and distributed in the periderm, as observed by previous studies [18,27,28].

### 2.2. De Novo Assembly of the P. ginseng Transcriptome

According to the chemical results, we focused on the transcriptome aspects of those three types of tissues: periderm, cortex, and stele, especially the periderm. Illumina Hiseq sequencing technology was employed, and RNA from the same 15 samples analyzed by high-performance liquid chromatography (HPLC) was used for RNA-seq. A total of 315,542,368, 318,145,782, and 320,110,684 raw reads were generated for the periderm, cortex, and stele, respectively. Each replication was represented by over 50 million reads with the highest 84 million reads, which is sufficient for the quantitative analysis of gene expression. After filtering, 306,370,476, 310,618,160, and 312,236,368 high-quality reads with Q30 > 94.6% were generated (Table S2), with an average length of 9,208,092,085 bp (Table 1). All 929,225,004 high-quality 150-bp reads were used for de novo assembly. In total 91,092 contigs > 500 bp in length were obtained, with an average contig size of 854 bp. A total of 182,881 unigenes were assembled, with an N50 contig size of 1374 bp (Table 1).

Based on a homology search with a cutoff E-value of 10^−5^ against public databases, including the NCBI non-redundant protein (Nr), Swiss-Prot, Gene Ontology (GO), and Kyoto Encyclopedia of Genes and Genomes (KEGG) databases, a total of 90,104 (49.3%) unigenes were annotated as at least one significant match in the above-mentioned databases. Among the 182,881 unigenes matched 85,928 (47.0%) unigenes currently deposited in the Nr database. Similarly, 78,250 unigenes, 63,817 unigenes, and 49,220 unigenes showed significant homologs to Swiss-Prot, GO terms, and KEGG passway, respectively. About half of the unigenes were not previously generated or deposited into public databases for *P. ginseng*, suggesting some of which may be tissue-specific, expressed in ginseng root (Table S3). The large quantity of novel unigenes identified in this study provides a powerful resource for further ginseng research, as well as the interpretation for the heterogeneous distribution of ginsenosides in ginseng root.

### 2.3. Functional Analysis of Unigenes

Additionally, GO terms were assigned for the unigene sets from three tissues. Under the three major GO categories of molecular function, cellular component, and biological process, these GO terms were further classified into 48 subcategories. A total of 246,880 GO terms were assigned for the periderm, 219,803 GO terms for the cortex, and 171,929 GO terms for the stele, indicating that a number of unigenes have two or more GO terms (Table S4). The majority of assigned GO terms were biological processes, followed by cellular components and molecular function categories. Catalytic and protein binding were the most abundant GO terms within the molecular function category. Considering the biological process category, the most dominant subcategories were in response to metabolic and cellular processes. ”Cell” and “cell part” were the most highly represented terms in the cellular component category. On the whole, the unigene sets from different tissues showed a very similar GO term profile of functional categorization (Figure S2, Table S4).

### 2.4. Distinct Transcriptome in Ginseng Main Root Tissues

**Figure 2:**
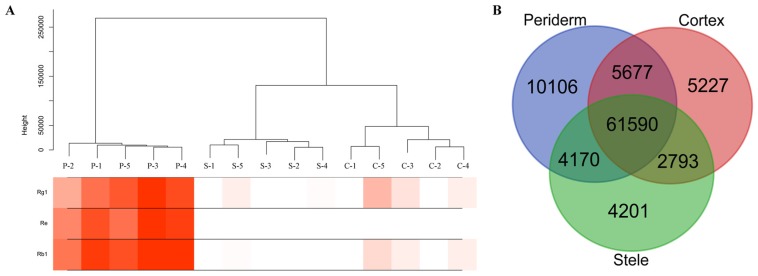
Clustering analysis of expression genes in ginseng main root tissues. (**A**) Clustering tree for expression genes in ginseng main root tissues. The leaves of the tree correspond to the three tissues with five duplicates. The color bands beneath the tree represent the relative content of the ginsenoside-Rg1, ginsenoside-Re, and ginsenoside-Rb1, with red indicating high values. P, C, and S refer to the periderm, cortex, and stele, respectively. (**B**) Venn diagram showing the overlap between the expression genes of each root tissue.

To investigate to what extent genes and biological processes are shared among ginseng main root tissues, we compared their expression genes and characterized the expression relationships among them. For the RNA-seq data, we calculated the expression level of each gene using FPKM (fragments per kilobase of exon model per million mapped reads). A total of 93,764 expressed genes remained from the transcriptome data, with 81,543 expressed in the periderm, 75,287 and 72,757 genes expressed in the cortex and stele, respectively (Table S5). All samples were assigned into three distinct categories by their relative expression profiles. The expression pattern of genes in the cortex was closer to that of the stele than to that of the periderm (Figure 2A). It appeared that the number of tissue-specific expressed unigenes varied among the three tissues, and a large overlap existed between them. From 4201 to 10,106 unigenes were observed to specifically express at one of the three tissues, with the periderm having the most tissue-specific expressed genes, followed by the cortex and stele, respectively. Of the unigenes, 61,590 (65.7%) were shared among the three tissues (Figure 2B).

Next, we calculated the statistics of the differential expression genes (DEGs) between two tissues with fold change (FC) ≥ 2 and the significant false discovery rate (FDR) ≤ 0.05. Comparing the periderm with the cortex and stele, a total of 3116 and 3311 DEGs were found, respectively. Comparing the cortex and stele, a total of 772 DEGs were found (Table S6). Collectively, the transcriptional profiles of periderm were clearly distinct from both the cortex and stele, supporting the notion that both of these are functionally different. Based on the GO enrichment analysis, the majority of DEGs between the periderm and cortex, as well as the periderm and stele, were mainly involved in single-organism and carbohydrate metabolic processes (Dataset S1). The commonalities and differences of expressed unigenes from the three tissues reflect evolutionarily-conserved programs and tissues that have physiological functions or responses to environmental conditions [41].

**Figure 3:**
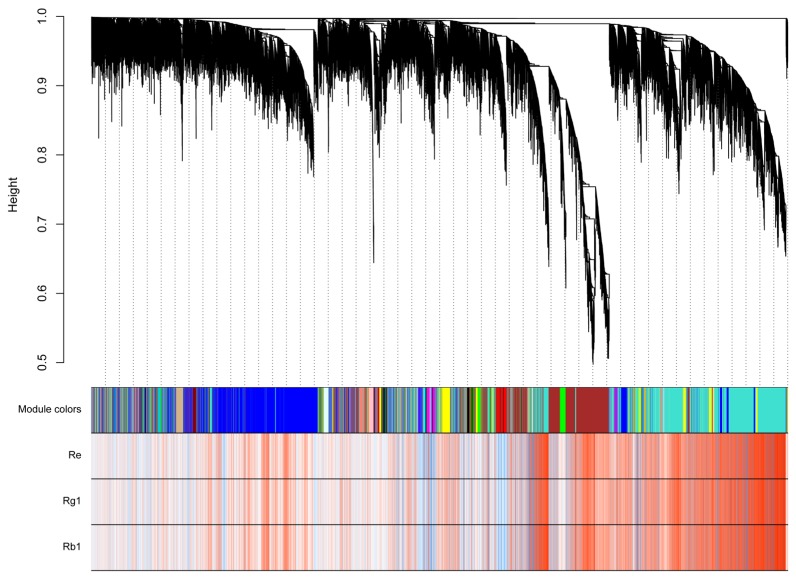
Co-expression analysis with 39,493 genes through the WGCNA. The modules corresponding to the branches are represented by colors in the first color band underneath the tree, and the remaining color bands reveal the correlation between transcripts and ginsenoside-Rg1, ginsenoside-Re and ginsenoside-Rb1. The red module indicates a highly positive correlation with the corresponding gene, the white module denotes a weak correlation, and the blue module represents a highly negative correlation.

A total of 39,493 genes from annotated unigenes were filtered with an average FPKM > 2 for each tissue. Then the hierarchical cluster tree was constructed with those genes through the weighted gene co-expression network analysis (WGCNA), and the content of ginsenoside-Rg1, ginsenoside-Re, and ginsenoside-Rb1 were considered as the weighted factors. All genes were grouped into 27 modules, of which 21,178 genes were positively correlated with ginsenosides (correlation coefficient > 0), and the most correlated module contained 9582 genes. This leads to the conclusion that the complex mechanisms are involved in ginsenoside synthesis and regulation (Figure 3, Table S7).

### 2.5. Analysis of Triterpenoid Saponins Metabolism

In order to improve the understanding of the regulatory networks of ginsenoside synthesis, accumulation, and transportation in different tissues, gene-encoding enzymes involved in ginsenoside biosynthesis and modification were investigated. A total of 24 genes (192 transcripts) encoding all of the known enzymes involved in ginsenoside skeleton biosynthesis through the mevalonate (MVA) and methylerythritol phosphate (MEP) pathways were identified in our dataset by BLAST search and motif finding. All of these enzyme genes were present in multiple copies and isoforms (ranging from two to 29 copies), which resulted in a wider variety of regulatory control of secondary metabolism biosynthesis in the plant (Table S7). We also identified candidates for major downstream genes, with which different ginsenoside precursors are cyclized and hydroxylated, including six farnesyl diphosphate synthase (FPS), 12 squalene epoxidases (SEs), seven beta-amyrin synthases (Beta-ASs), five oleanolic acid synthases (OASs), and two dammarendiol synthases (DDSs). Additionally, five protopanaxadiol synthases (PPDSs), and three protopanaxatriol synthases (PPTSs) were found in our data set (Table S8).

**Figure 4:**
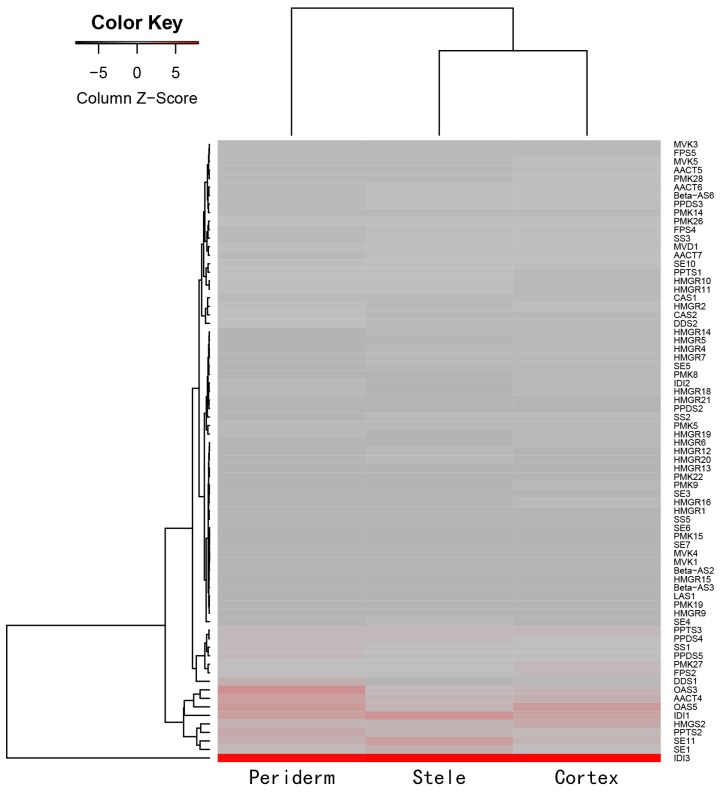
Heatmap of 73 MVA pathway and downstream genes involved in ginsenoside biosynthesis in ginseng main root tissues, this was constructed using an average FPKM value of five duplicates (FPKM ≥ 2).

Furthermore, we extracted the expression data of the 192 transcripts with FPKM ≥ 2 and characterized the expression relationships by heatmap analysis (Figure 4, Table S8). Most transcripts in the MVA pathway were variably expressed in the three tissues. Overall, the expression tendency of the transcripts was similar between the stele and cortex. Among highly-abundant transcripts, transcripts encoding acetyl-CoA C-acetyltransferase (AACT), OSA, DDS, PPDS, and PPTS were more highly expressed in the periderm than in the cortex and stele. The results showed the coordinate expression and coordinate regulation among gene families involved in ginsenoside biosynthesis. Transcripts encoding 3-hydroxy-3-methylglutaryl-CoA synthase (HMGS) and OSA were more highly expressed in the cortex. Three transcripts encoding SE were more highly expressed in the stele (Figure 4, Table S8). The similarities and differences of expression profiles may be responsible for the uneven distribution of ginsenosides in ginseng root. Similar findings were presented for the MEP pathway, another ginsenoside biosynthesis pathway when MVA was blocked. Among highly-abundant transcripts, transcripts encoding isoprenoid synthase-containing protein D (IspD, two transcripts) and isopentenyl diphosphate isomerase (IDI, two transcripts) were all highly expressed in the three tissues. Transcripts encoding 1-deoxy-d-xylulose-5-phosphate synthase (DXS), isoprenoid synthase-containing protein H (IspH), and IspD were more highly expressed in the periderm than in the cortex and stele. Transcripts encoding DXS, 1-deoxy-d-xylulose-5-phosphate reductoisomerase (DXR), isoprenoid synthase-containing protein F (IspF), IspH (three transcripts), and isoprenoid synthase-containing protein gcpE (IspG/gcpE) were more highly expressed in the cortex. Transcripts encoding DXS (two transcripts) and isoprenoid synthase-containing protein E (IspE) were more highly expressed in the stele (Figure S3, Table S8).

A total of 290 UGTs, which may catalyze glycosylation in the last step of ginsenosides biosynthesis, were found in the ginseng main root transcriptome. The abundance of UGTs in the periderm was highly variable compared with that in the cortex and stele. Multiple highly-expressed UGTs and a variety of expression profiles suggested complex regulatory mechanisms of various ginsenoside biosynthesis in different tissues/species (Figure S4, Table S9). This indicative data provides a strong basis and reference for further functional verification of candidate UGTs.

## 3. Discussion

Herbgenomics, an emerging discipline, aims to explore the genetics and biology of herbs at the genome level, and has been extensively applied in herb-related biological research [42]. The research results will lay the foundation for elucidating the biosynthesis and regulation for active compounds of herbs, and will then promote the screening of plant drugs and herb synthetic biology research, and will also accelerate the breeding of fine varieties of medicinal plants. It is now broadening the interpretation of biological mechanisms for most traditional medicines [43,44,45,46,47,48,49,50]. In this study, we integrated the analysis of chemicals and transcriptomes simultaneously in ginseng root for a comprehensive characterization for ginsenoside biosynthesis.

As mentioned earlier, the accumulation and distribution of ginsenosides is species- and tissue-specific, and even displays heterogeneous distribution in ginseng root [18,27,28]. In agreement with previous research results, we also found that multiple ginsenosides were mainly distributed and stored in the periderm. This uneven accumulation pattern of secondary metabolites potentially impacts root survival under the complex soil environment, including potential plant diseases and insect pests [33,34].

Meanwhile, to maximize biological diversity, the same 15 samples of the HPLC experiment were all used for profiling the differential transcriptome among the three tissues. In this study, an Illumina Hiseq sequencing platform with increased sequencing throughput was used to enable more transcript information to be obtained. More than 929 million high-quality reads were generated just from the main root and resulted in 182,881 unigene de novo assemblies. Furthermore, about half of the unigenes were identified as novel unigenes. The data size generated in our study is beyond the latest transcriptome studies for ginseng root [6,35,37,38,51] and refreshes the transcriptome data for ginseng once again. These numerous unigenes provide a powerful resource for further study of functional genomics in *P. ginseng*.

The transcriptome comparison analysis demonstrated the commonalities and differences of expressed unigenes among three root tissues. The semblable GO term profile of functional categorization assigned to unigenes from the periderm, cortex, and stele may be because the three tissues are from the root, resulting in a larger proportion of overlapping genes. In addition, we focused on the relationship between ginsenoside content and expressed unigenes. The results showed that a large number of unigenes may act on ginsenoside biosynthesis, and indicated the complex mechanisms involved in ginsenoside synthesis and regulation in different tissues.

The large amount of transcriptome data obtained in the present study promotes the identification of genes related to ginsenoside biosynthesis. We identified candidate MVA and MEP pathway genes involved in ginsenoside synthesis. On the whole, the expression profiles of the transcripts were varied among the three tissues. HMGR has been considered as the first rate-limiting enzyme of ginsenoside synthesis. Kim et al. identified two copies of HMGR and considered one copy (PgHMGR2) may be responsible for the distinct age-dependent ginsenoside accumulation in the root [52]. Jayakodi et al. found three HMGR transcripts, one of which was highly expressed in the main root body [38]. In the present study, 22 HMGR transcripts were identified in three root tissues: of which six transcripts were more highly expressed in the periderm than in the other two tissues, and 11 transcripts were also identified as DEGs in comparison between the two tissues. Several transcripts encoding AACT, OSA, and DDS were also more highly expressed in the periderm. Jayakodi et al. found two CYP450 genes encoding PPDS (CYP716A47) and PPTS (CYP716A53V2), which were highly expressed in the main root body of one-year-old samples and the rhizomes of six-year-old samples, respectively [38]. Here, we identified five PPDS and three PPTS from the unigene dataset: of which, three PPDS and two PPTS were more highly expressed in the periderm. For 290 transcripts encoding UGTs, 195 UGTs (67.2%) were more highly expressed in the periderm, 83 UGTs were also identified as DEGs in comparing the periderm with the cortex and stele. The number of transcripts encoding putative UGTs is larger than that previously analyzed [6,35,37,38,51], and that seven UGTs and one UGT were specifically expressed in the periderm and stele, respectively (FPKM ≥ 2) (Table S9). These highly-expressed transcripts in the periderm, and the differential expression profiles among different tissues, may be responsible for the uneven accumulation of ginsenosides, and provide strong candidates for further study of functional genomics in *P. ginseng*.

## 4. Materials and Methods

### 4.1. Plant Material

The fresh *P. ginseng* roots aged four years were sampled from Jingyu County in Jilin Province, China (August 2016). Each main root was carefully peeled into three tissues, namely the periderm, cortex, and stele (xylem and medulla), and five duplicates generated 15 samples (Figure S5). Each sample was divided into two, one was immediately frozen in liquid nitrogen and stored at −80 °C for transcriptome profiling by RNA-seq, and another was stored at −20 °C for ginsenoside investigation by HPLC until use.

### 4.2. HPLC Analysis

A slightly modified ultrasonic extraction of a previously described protocol was employed for the extraction of ginsenosides [53]. Briefly, approximately 200 mg of each sample was crushed and soaked in 2 mL of methanol overnight. After ultrasound lysis at room temperature for 30 min (500 W, 40 kHz), the extracts were centrifuged for 5 min (14,000 r·min^−1^) and the supernatant was transferred to another test tube. Ultrasound lysis was repeated again, then merged with two supernatants and dried methanol at 100 °C in the water bath. The residue was subsequently dissolved in 2 mL of methanol. This solution was filtered through a 0.45 μm organic micropore membrane prior to use.

Analyses of target compounds were carried out with an Agilent 1260 series HPLC system. An Eclipse XDB-C18 column (5 μm, 4.6 mm ID × 250 mm) was used for optimal separation. The mobile phase was formed from acetonitrile (solvent A) and water (solvent B) with gradient elution as follows, according to the Pharmacopoeia of the People’s Republic of China (2015 Edition): 0–35 min, 19% A; 35–55 min, 19–29% A; 55–70 min, 29% A; 70–100 min, 29–40% A, at a flow rate of 1.0 mL/min. The column temperature was set to 35 °C. Detection wavelength was 203 nm and the injection volume was 10 μL. Quantitative analysis was performed using an external standard method.

### 4.3. RNA-Seq

Total RNA was extracted from the pulverized sample with a polysaccharide plant polyphenols quick RNA extraction kit (Cat. No. RP3202, BioTeke Corporation, Beijing, China, http://www.bioteke.com) under the protocols of the manufacturer’s instructions. All RNA samples were treated with Dnase I on column using Pure Link™ DNase (Cat. No. 12185-010, Invitrogen, Carlsbad, CA, USA, http://www.thermofisher.com). The quality and quantity of RNA were accessed and determined by a bioanalyzer (Agilent Technologies, Santa Clara, CA, USA) and agarose gel electrophoresis. Transcriptome libraries for RNA-seq were prepared with 2 μg total RNA following the Illumina TruSeq RNA prep protocols, including selection for the cDNA target fragments from the 200–300 bp size range. After PCR amplification for 15 cycles and quantification, the paired-end of 150 bp libraries for the 15 samples were sequenced by Illumina HiSeq 4000 (Illumina, Inc., San Diego, CA, USA).

### 4.4. Transcriptome Analysis

The raw sequencing reads were processed by Skewer and FastQC, and the clean reads of each sample were obtained. De novo assembly from the clean reads of all 15 samples was conducted using Trinity with default parameters [54,55]. For gene function annotation, the representative sequence was defined with the transcript encoding the longest protein sequence for each gene. Homology similarity searching against databases was performed through BLASTX with the best similar hit of an E-value < 1e^−5^, including NCBI (ftp://ftp.ncbi.nih.gov/blast/db) and Swiss-Prot (http://www.uniprot.org/). Classification of protein function was searched against the GO database (http://www.geneontology.org/) and KEGG database (http://www.genome.jp/tools/kaas/).

Cufflinks (http://cole-trapnell-lab.github.io/cufflinks/) was employed to estimate the transcript abundance [56]. The FPKM was calculated to quantify the expression level and identify DEGs among the different samples. The identification of DEGs was performed using the following criteria: FC ≥ 2 and FDR ≤ 0.05. The directed acyclic graph of GO enrichment analyses was performed through the Goatools (https://github.com/tanghaibao/Goatools). The hierarchical clustering analysis of the expression profiles was performed using the hclust command in R and the default complete linkage method. The R package WGCNA was used to identify the co-expression modules, which were plotted using Cytoscape for visualization. The heatmaps were constructed using the R programming language and software.

### 4.5. Data Availability

The transcriptome data for replicate-2 and -5 have been deposited at NCBI under BioProject PRJNA369187. The remaining transcriptome data (replicate-1, -3, and -4) were deposited at NCBI under BioProject PRJNA381509.

## 5. Conclusions

This study is the first report to investigate the transcriptome differences for ginseng main root at the tissue level using RNA-seq and to answer the biological questions related to the uneven distribution of ginsenosides. On the basis of RNA-Seq analysis, this study implemented a comprehensive characterization for ginsenoside biosynthesis in ginseng root. It was found that the accumulation and distribution of ginsenosides were closely correlated with genes involved in their biosynthesis and regulation, which displayed a complex mechanisms. This genetic resource will help to improve the interpretation of ginsenoside synthesis, accumulation, and transportation.

## Figures and Tables

**Table 1:** Summary of the transcriptome data and the assembly results for ginseng main root tissues.

Item	No. of Sequences
High-quality reads	929,225,004
Average length (bp)	9,208,092,085
No. of contig > 500 bp	91,092
Total unigenes	182,881
N50 contig size (bp)	1374
Total assemble bases (bp)	156,275,266

## Supplementary Materials

See the word file of “The list of supplementary materials”, all supplementary materials are available online.

## Abbreviations

UGTs	UDP-glycosyltransferases
DEGs	Differential expression genes
MALDI-MS	Matrix-assisted laser desorption/ionization mass spectrometry
HPLC	High-performance liquid chromatography
NCBI Nr	NCBI non-redundant protein non-redundant protein
GO	Gene Ontology
KEGG	Kyoto Encyclopedia of Genes and Genomes
ORF	Open read frame
FPKM	Fragments per kilobase of exon model per million mapped reads
WGCNA	Weighted gene co-expression network analysis
MVA	Mevalonate
MEP	Methylerythritol phosphate
FPS	Farnesyl diphosphate synthase
SE	Squalene epoxidase
Beta-ASs	Beta-amyrin synthases
OASs	Oleanolic acid synthases
DDSs	Dammarendiol synthases
PPDSs	Protopanaxadiol synthase
PPTSs	Protopanaxatriol synthases
AACT	Acetyl-CoA *C*-acetyltransferase
HMGS	3-Hydroxy-3-methylglutaryl-CoA synthase
IspD	Isoprenoid synthase-containing protein D
IDI	Isopentenyl diphosphate isomerase
DXS	1-Deoxy-d-xylulose-5-phosphate synthase
IspH	Isoprenoid synthase-containing protein H
DXR	1-Deoxy-d-xylulose-5-phosphate reductoisomerase
IspF	Isoprenoid synthase-containing protein F
IspG/gcpE	Isoprenoid synthase-containing protein gcpE
IspE	Isoprenoid synthase-containing protein E
