# Supplementary material for: Comprehensive Characterization for Ginsenosides Biosynthesis in Ginseng Root by Integration Analysis of Chemical and Transcriptome
Source: Molecules. 2017 May 31;22(6):889. doi: 10.3390/molecules22060889 (PMC6152789; doi:10.3390/molecules22060889)
Supplement: Supplementary file 1 [file molecules-22-00889-s001.zip › molecules-192541-supplementary /The list of supplementary materials.docx]

**The list of supplementary materials:**

Supplementary Material 1: Figure S1. HPLC ginsenoside profile of *P. ginseng* three main root tissues. **A**. Ginsenosides standards; **B**. Periderm; **C**. Cortex; and **D**. Stele. Detection at 203 nm wavelength. Peaks: **1**. ginsenoside-Rg1; **2**. ginsenoside-Re; **3**. ginsenoside-Rf; **4**. ginsenoside-Rg2; **5**. ginsenoside-Rb1; **6**. ginsenoside-Rc; **7**. ginsenoside-Rb2; and **8**. ginsenoside-Rd.

Supplementary Material 2: Table S1. The contents of eight ginsenosides in ginseng main root tissues (mg/g). P, C, and S refer to the periderm, cortex, and stele, respectively.

Supplementary Material 3: Table S2. Statistics of transcriptome data for ginseng main root tissues. P, C, and S refer to the periderm, cortex, and stele, respectively.

Supplementary Material 4: Table S3. Homology and protein sequence similarity search of the unigene set.

Supplementary Material 5: Table S4. Statistics of GO analysis for ginseng main root tissues.

Supplementary Material 6: Figure S2. GO classifications for unigenes in periderm, cortex, and stele.

Supplementary Material 7: Table S5. The expression level of unigenes in 15 samples. P, C, and S refer to the periderm, cortex, and stele, respectively. Supplementary Material 8: Table S6. Statistics of differential expression unigenes between two tissues (FDR ≤ 0.05 and FC ≥ 2).

Supplementary Material 9: Dataset S1. A compressed file containing all GO enrichment analysis (TOP 20 terms of biological process) of DEGs and the directed acyclic graphs (TOP 10 terms of biological process). **A**. The results for downregulated unigenes in cortex vs. stele. **B**. The results for upregulated unigenes in cortex vs. stele. **C**. The results for downregulated unigenes in periderm vs. cortex. **D**. The results for upregulated unigenes in periderm vs. cortex. **E**. The results for downregulated unigenes in periderm vs. stele. **F**. The results for upregulated unigenes in periderm vs. stele.

Supplementary Material 10: Table S7. The merged colors and correlation coefficient for 39,493 genes in the co-expression analysis through the WGCNA. Supplementary Material 11: Table S8. Candidate 192 transcripts involved in ginsenoside biosynthesis and their expression level in 15 samples. Supplementary Material 12: Table S9. Candidate 290 transcripts encoding UGT protein and their expression level in 15 samples. The eight highlighted UGTs were transcripts specifically expressed in the periderm and stele (FPKM ≥ 2). Supplementary Material 13: Figure S3. Heatmap of transcripts involved in the ginsenoside biosynthesis MEP pathway in ginseng main root tissues, which was constructed using average FPKM values of five duplicates (FPKM ≥ 2). Supplementary Material 14: Figure S4. Heatmap of transcripts encoding UGT protein involved in ginsenoside biosynthesis in ginseng main root tissues, which was constructed using average FPKM values of five duplicates (FPKM ≥ 2).

Supplementary Material 15: Figure S5. The ginseng main root tissues used in this study. **a**. ginseng root cross section; **b**. periderm; **c**. cortex; and **d**. stele.
