# Supplementary figures and images for: Comprehensive Characterization for Ginsenosides Biosynthesis in Ginseng Root by Integration Analysis of Chemical and Transcriptome
Source: Molecules. 2017 May 31;22(6):889. doi: 10.3390/molecules22060889 (PMC6152789; doi:10.3390/molecules22060889)

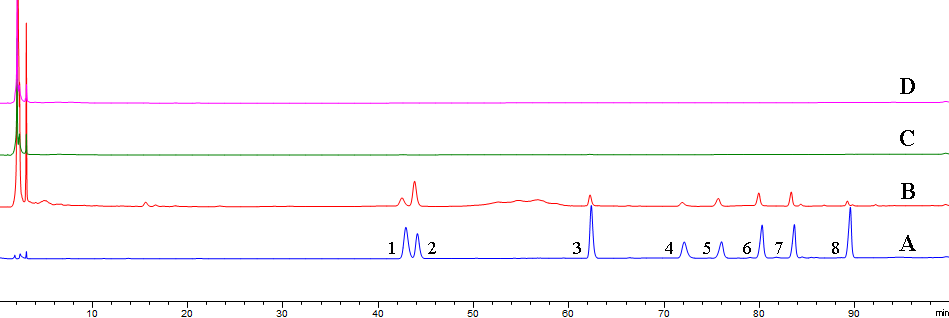

Supplement: Supplementary file 1 [file molecules-22-00889-s001.zip › molecules-192541-supplementary /Supplementary material 1-Figure S1.tif]

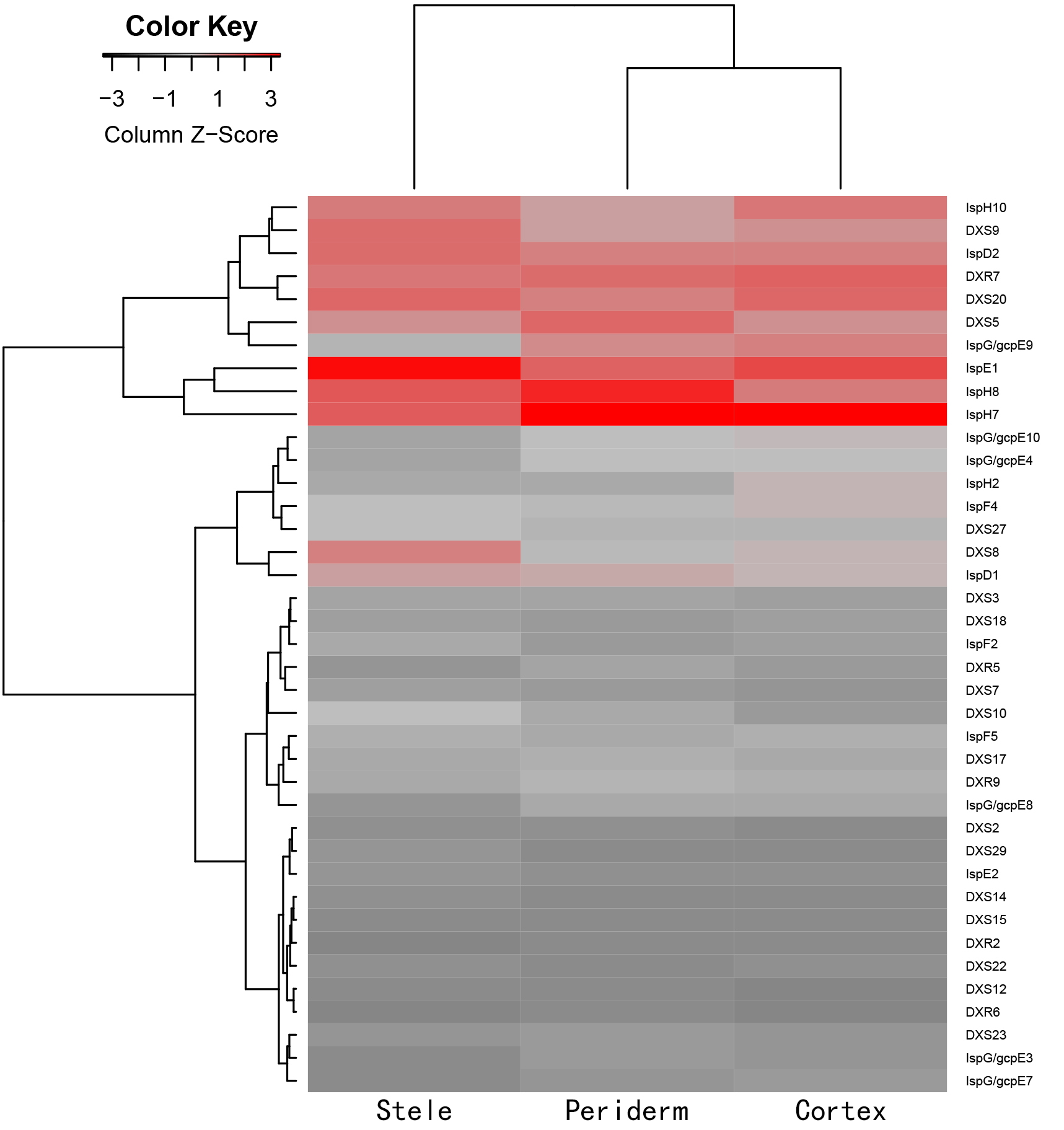

Supplement: Supplementary file 1 [file molecules-22-00889-s001.zip › molecules-192541-supplementary /Supplementary material 13-Figure S3.jpg]

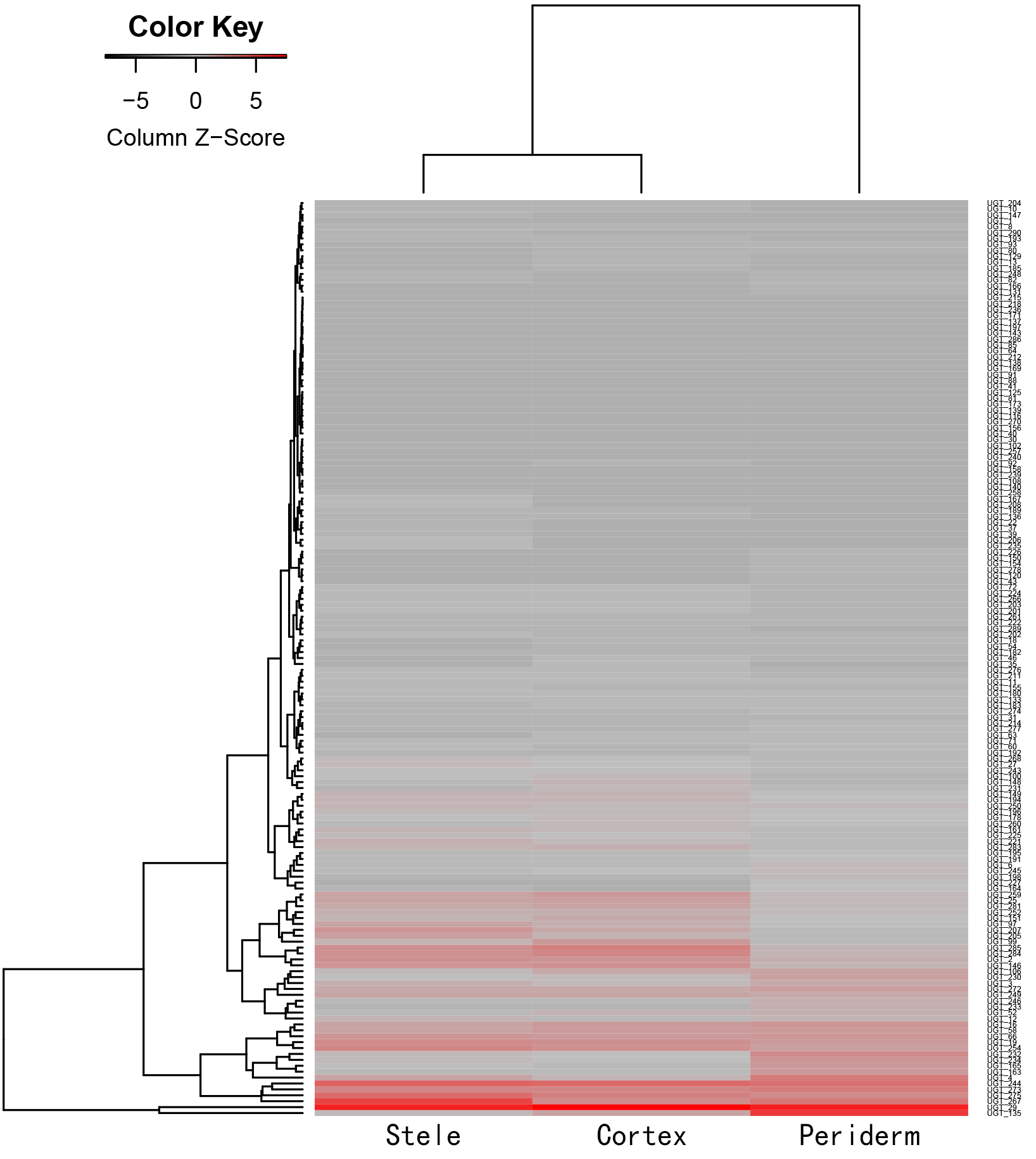

Supplement: Supplementary file 1 [file molecules-22-00889-s001.zip › molecules-192541-supplementary /Supplementary material 14-Figure S4.jpg]

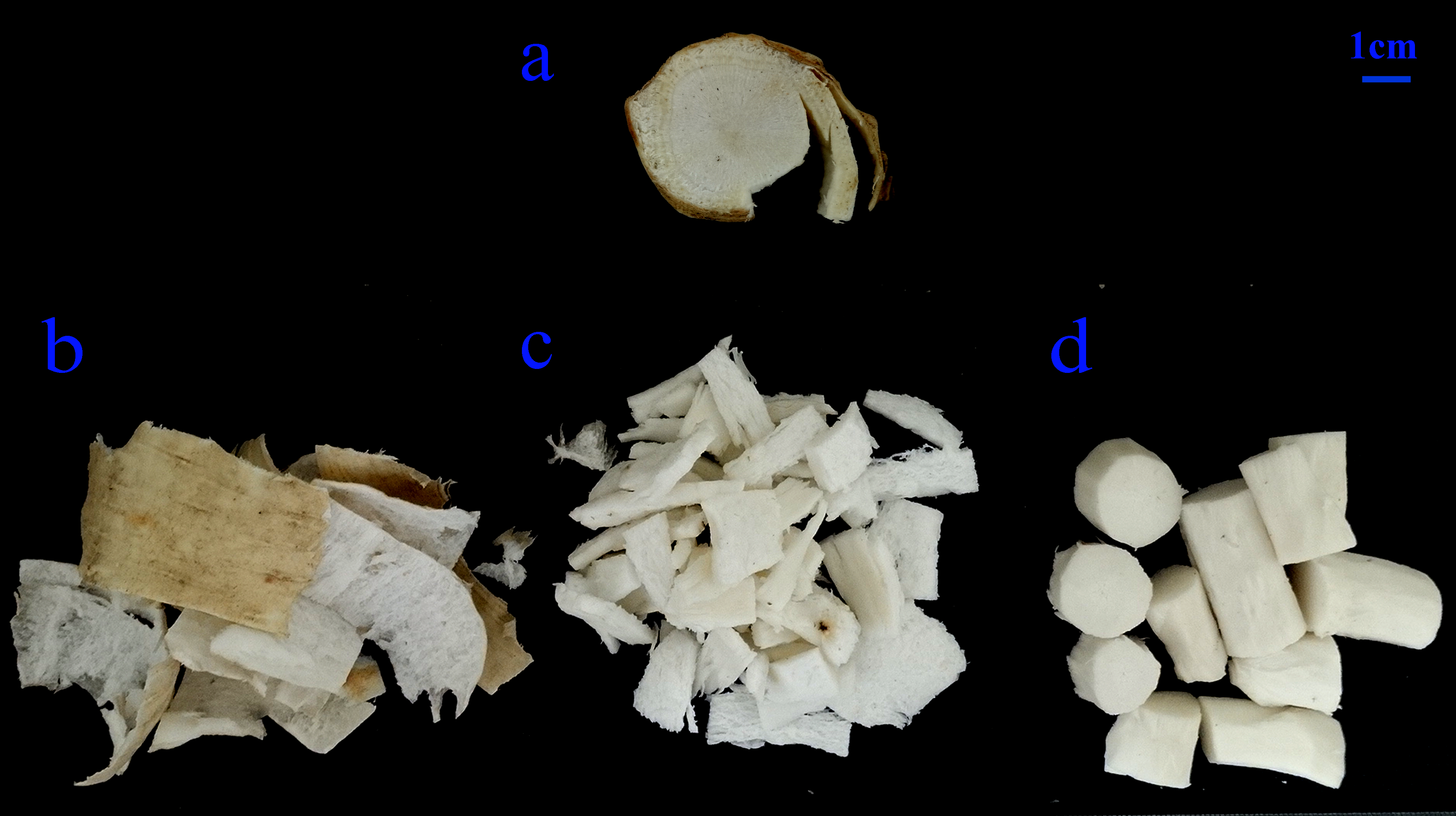

Supplement: Supplementary file 1 [file molecules-22-00889-s001.zip › molecules-192541-supplementary /Supplementary material 15-Figure S5.tif]

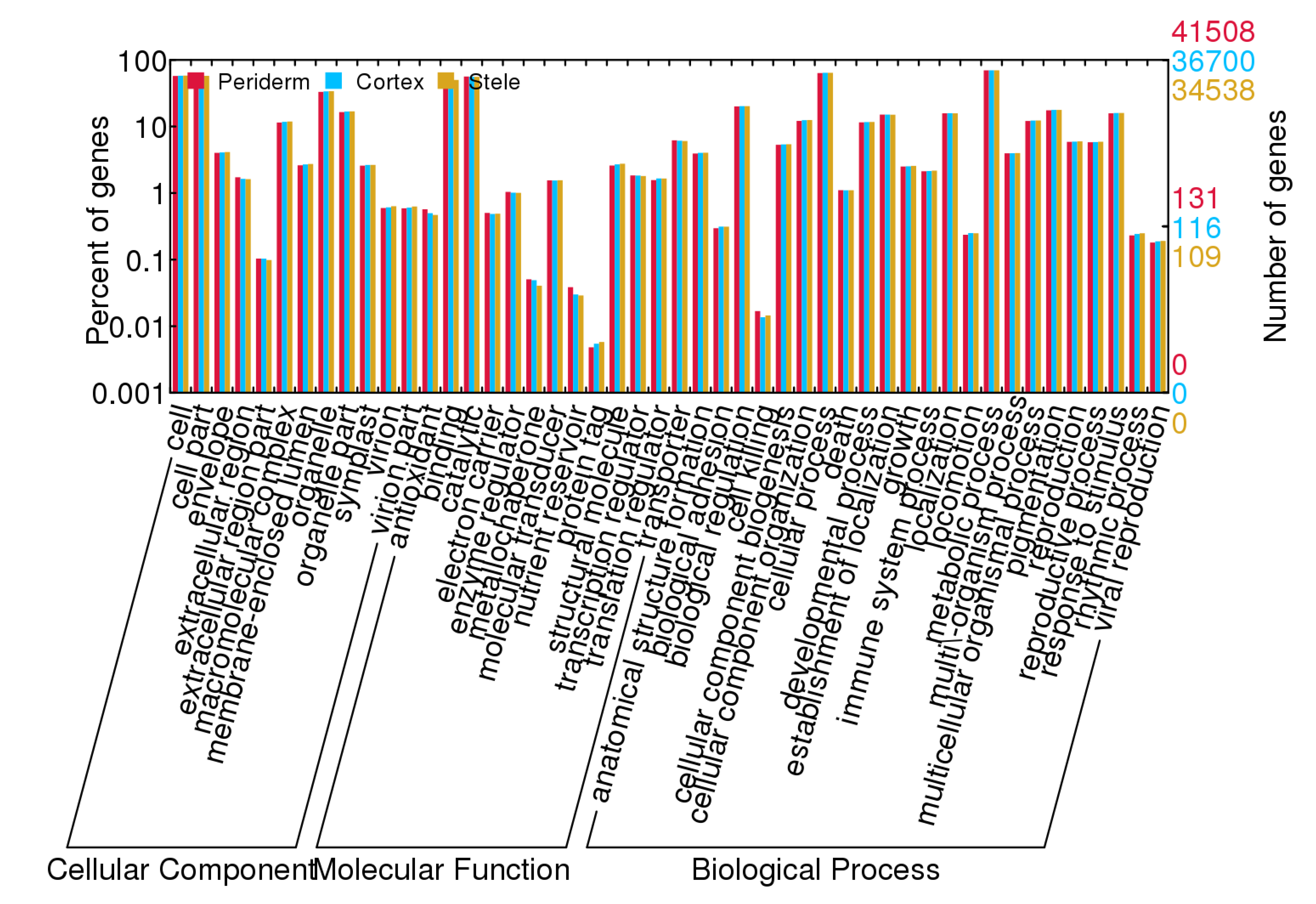

Supplement: Supplementary file 1 [file molecules-22-00889-s001.zip › molecules-192541-supplementary /Supplementary material 6-Figure S2.tif]

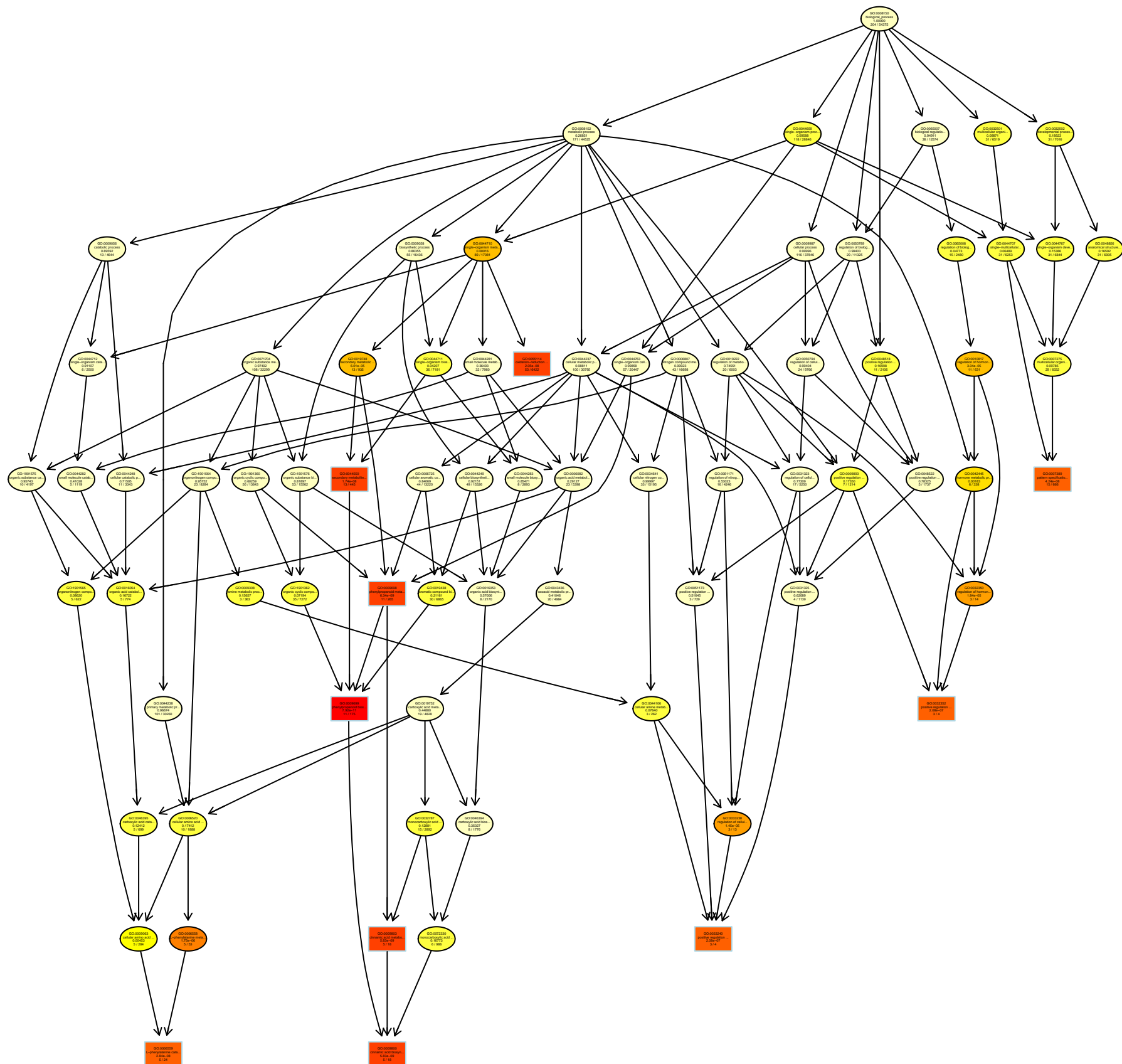

Supplement: Supplementary file 1 [file molecules-22-00889-s001.zip › molecules-192541-supplementary /Supplementary material 9-Dataset S1/A/CvsS_down_BPGO_classic_10_all.pdf]

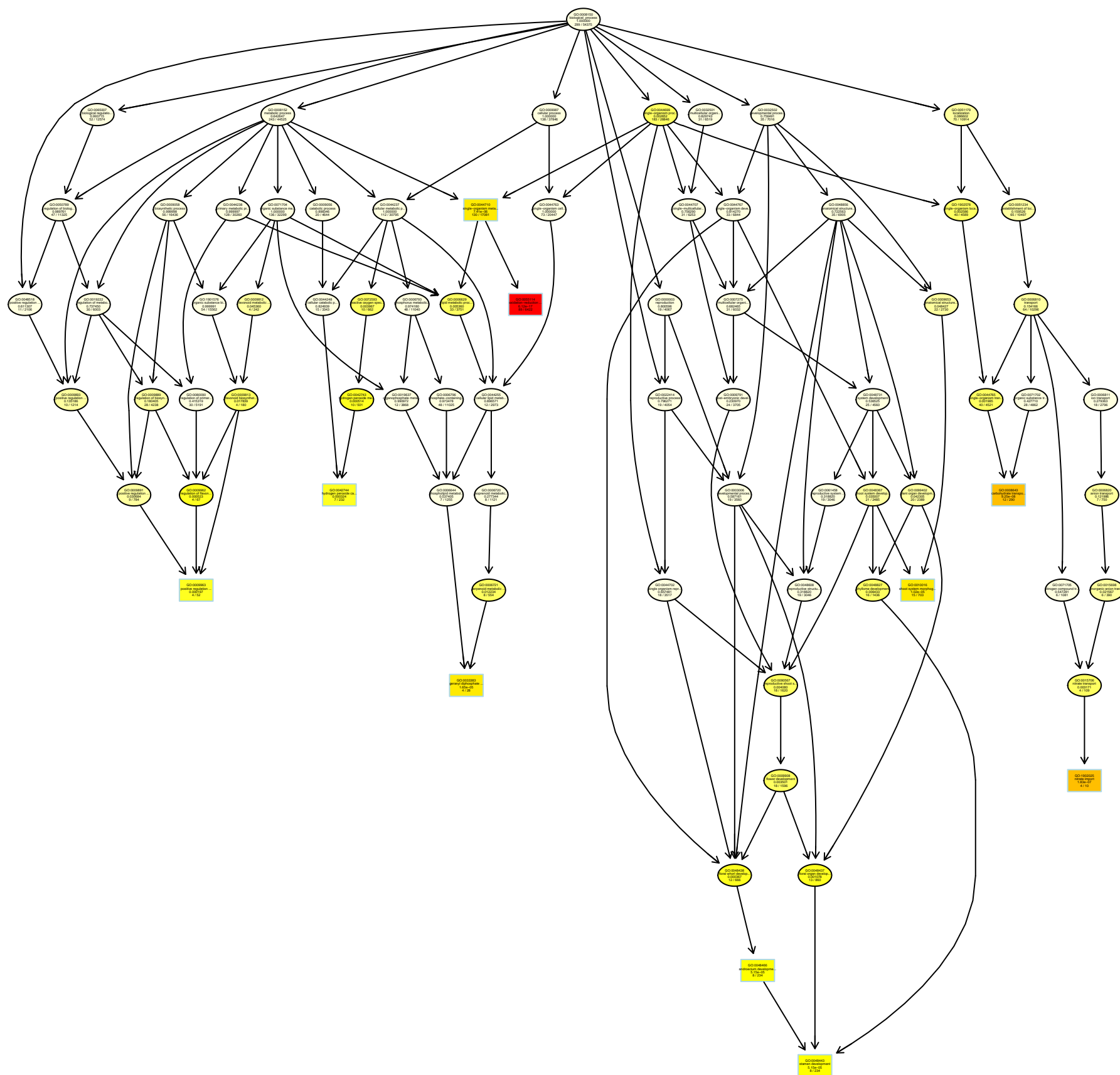

Supplement: Supplementary file 1 [file molecules-22-00889-s001.zip › molecules-192541-supplementary /Supplementary material 9-Dataset S1/B/CvsS_up_BPGO_classic_10_all.pdf]

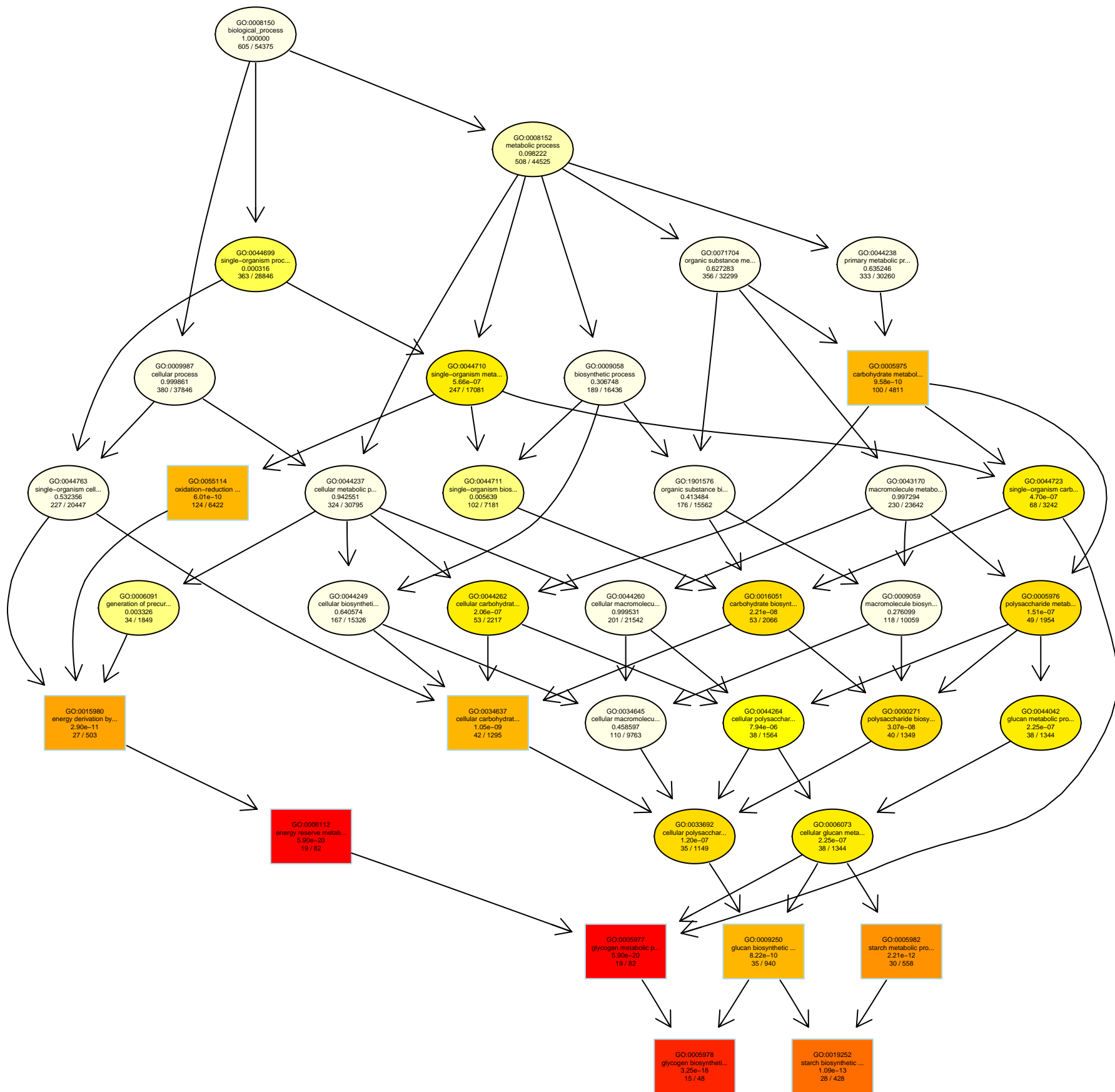

Supplement: Supplementary file 1 [file molecules-22-00889-s001.zip › molecules-192541-supplementary /Supplementary material 9-Dataset S1/C/PvsC_down_BPGO_classic_10_all.pdf]

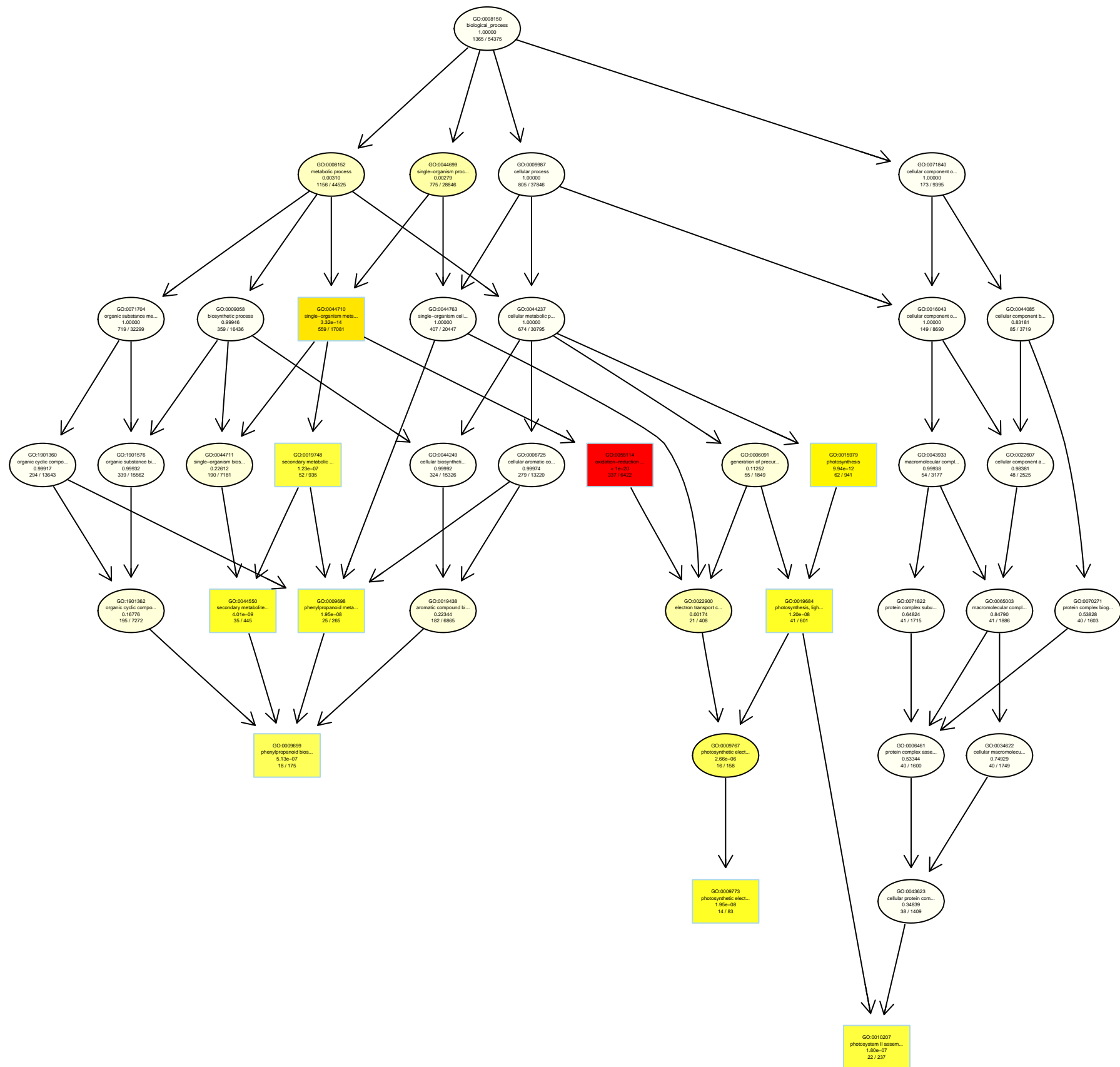

Supplement: Supplementary file 1 [file molecules-22-00889-s001.zip › molecules-192541-supplementary /Supplementary material 9-Dataset S1/D/PvsC_UP_BPGO_classic_10_all.pdf]

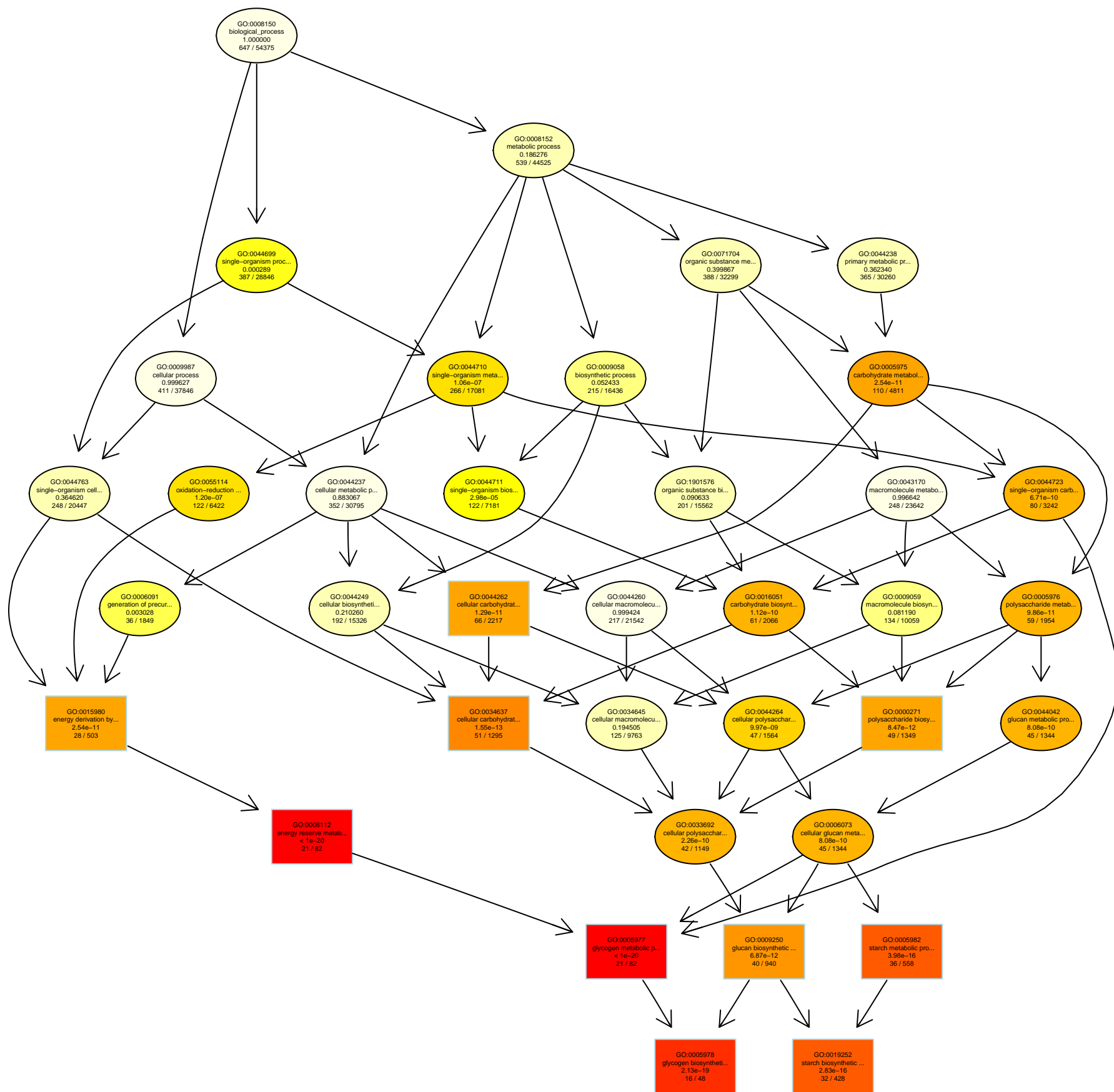

Supplement: Supplementary file 1 [file molecules-22-00889-s001.zip › molecules-192541-supplementary /Supplementary material 9-Dataset S1/E/PvsS_down_BPGO_classic_10_all.pdf]

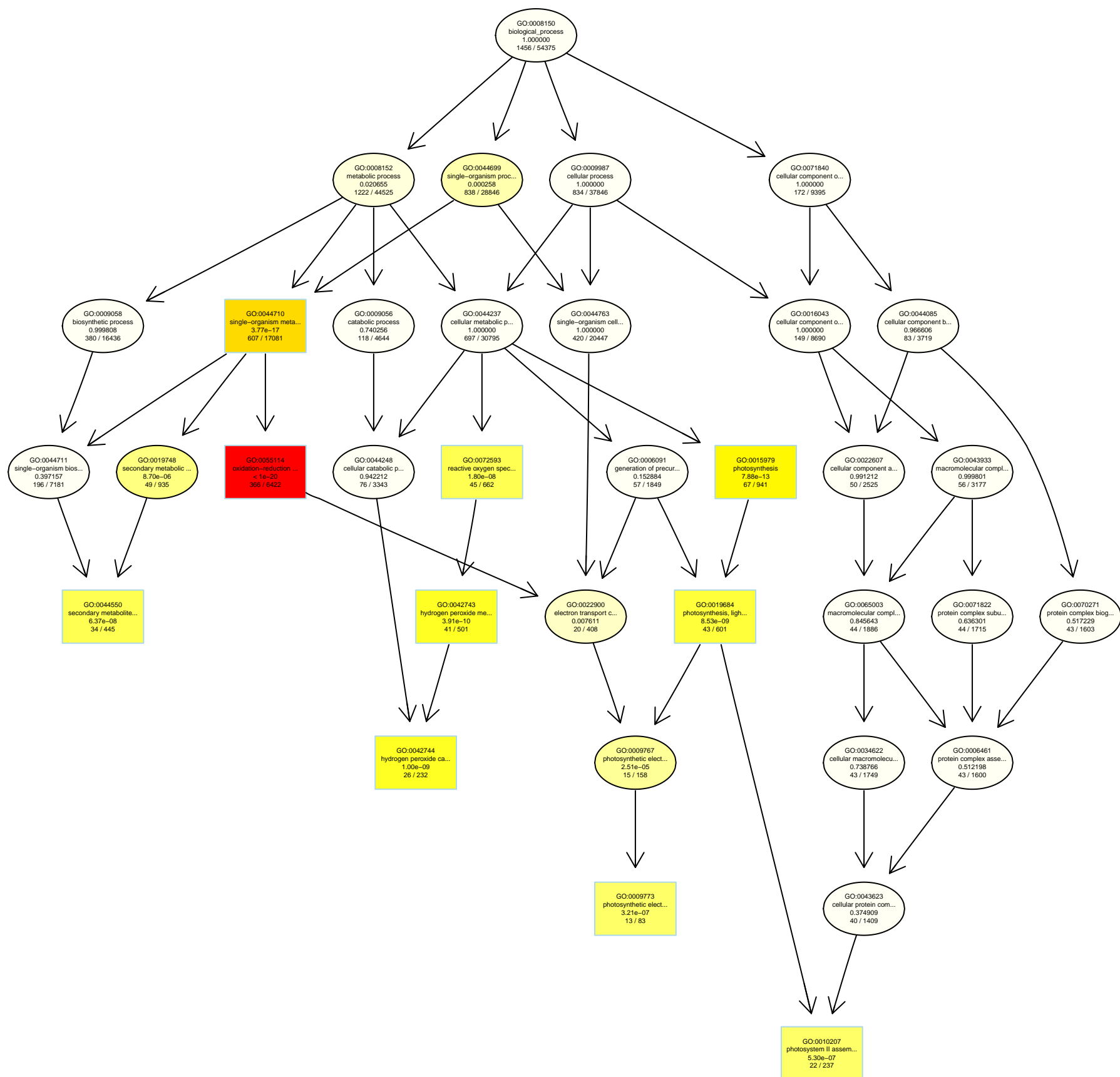

Supplement: Supplementary file 1 [file molecules-22-00889-s001.zip › molecules-192541-supplementary /Supplementary material 9-Dataset S1/F/PvsS_up_BPGO_classic_10_all.pdf]
