# Supplementary material for: Comprehensive Characterization for Ginsenosides Biosynthesis in Ginseng Root by Integration Analysis of Chemical and Transcriptome
Source: Molecules. 2017 May 31;22(6):889. doi: 10.3390/molecules22060889 (PMC6152789; doi:10.3390/molecules22060889)
Supplement: Supplementary file 1 [file molecules-22-00889-s001.zip › molecules-192541-supplementary /Supplementary material 2-Table S1.doc]

**Table S1.** The contents of eight ginsenosides in ginseng main root tissues (mg/g). P, C, and S refer to the periderm, cortex, and stele, respectively.

| **Tissue** | **Replicates** | **Rg1** | **Re** | **Rf** | **Rg2** | **Rb1** | **Rc** | **Rb2** | **Rd** | **Total** |
| --- | --- | --- | --- | --- | --- | --- | --- | --- | --- | --- |
| Periderm | P-1 | 2.455±0.012 | 8.403±0.180 | 2.385±0.070 | 2.420±0.070 | 3.927±0.113 | 4.677±0.019 | 3.848±0.108 | 1.199±0.038 | 29.314±0.587 |
|  | P-2 | 1.513±0.033 | 5.868±0.087 | 1.220±0.030 | 1.569±0.020 | 2.803±0.036 | 1.972±0.022 | 1.713±0.055 | 0.461±0.004 | 17.119±0.246 |
|  | P-3 | 3.490±0.263 | 9.774±1.302 | 1.434±0.025 | 1.449±0.017 | 4.100±0.091 | 5.838±0.367 | 4.547±0.095 | 0.954±0.024 | 31.585±2.183 |
|  | P-4 | 3.041±0.242 | 9.115±0.302 | 1.754±0.049 | 2.127±0.047 | 4.071±0.095 | 4.801±0.128 | 3.725±0.078 | 0.992±0.023 | 29.626±0.966 |
|  | P-5 | 2.853±0.086 | 6.845±0.014 | 1.957±0.032 | 1.724±0.006 | 3.533±0.060 | 3.053±0.136 | 2.837±0.050 | 0.693±0.008 | 23.495±0.392 |
|  | Average | 2.670±0.746 | 8.001±1.615 | 1.750±0.455 | 1.858±0.405 | 3.687±0.543 | 4.068±1.538 | 3.334±1.091 | 0.860±0.286 | 26.228±5.921 |
| Cortex | C-1 | 0.157±0.002 | 0.077±0.000 | 0.130±0.001 | - | 0.137±0.002 | 0.137±0.001 | 0.109±0.004 | 0.156±0.003 | 0.903±0.011 |
|  | C-2 | 0.188±0.002 | 0.079±0.002 | 0.151±0.000 | - | 0.153±0.002 | 0.131±0.001 | 0.101±0.000 | 0.157±0.015 | 0.959±0.011 |
|  | C-3 | 0.671±0.023 | 0.105±0.036 | 0.226±0.005 | - | 0.445±0.009 | 0.223±0.003 | 0.181±0.005 | 0.173±0.014 | 2.023±0.068 |
|  | C-4 | 0.454±0.007 | 0.115±0.004 | 0.244±0.002 | - | 0.464±0.003 | 0.201±0.000 | 0.165±0.000 | 0.186±0.004 | 1.829±0.012 |
|  | C-5 | 1.292±0.001 | 0.201±0.041 | 0.477±0.010 | 0.096±0.003 | 0.875±0.011 | 0.295±0.003 | 0.230±0.004 | 0.258±0.003 | 3.724±0.031 |
|  | Average | 0.552±0.464 | 0.115±0.051 | 0.246±0.138 | 0.096±0.003 | 0.415±0.300 | 0.197±0.068 | 0.157±0.053 | 0.186±0.042 | 1.888±1.143 |

**Table 1.** (*Continued*)

| **Tissue** | **Replicates** | **Rg1** | **Re** | **Rf** | **Rg2** | **Rb1** | **Rc** | **Rb2** | **Rd** | **Total** |
| --- | --- | --- | --- | --- | --- | --- | --- | --- | --- | --- |
| Stele | S-1 | 0.140±0.028 | 0.073±0.001 | 0.109±0.001 | - | 0.095±0.000 | 0.119±0.001 | 0.094±0.003 | 0.161±0.001 | 0.770±0.004 |
|  | S-2 | 0.159±0.002 | 0.079±0.002 | 0.130±0.000 | - | 0.115±0.001 | 0.123±0.003 | 0.095±0.001 | 0.177±0.005 | 0.876±0.013 |
|  | S-3 | 0.176±0.001 | 0.081±0.001 | 0.111±0.000 | 0.108±0.076 | 0.128±0.002 | 0.126±0.001 | 0.097±0.000 | 0.150±0.000 | 0.922±0.072 |
|  | S-4 | 0.219±0.007 | 0.151±0.010 | 0.144±0.001 | 0.098±0.007 | 0.153±0.002 | 0.134±0.002 | 0.105±0.000 | 0.161±0.004 | 1.165±0.012 |
|  | S-5 | 0.444±0.007 | 0.157±0.004 | 0.216±0.001 | 0.090±0.000 | 0.237±0.000 | 0.146±0.003 | 0.112±0.002 | 0.166±0.001 | 1.523±0.070 |
|  | Average | 0.228±0.124 | 0.108±0.042 | 0.142±0.044 | 0.099±0.009 | 0.145±0.056 | 0.130±0.011 | 0.101±0.008 | 0.163±0.010 | 1.051±0.301 |
