# Supplementary material for: Comprehensive Characterization for Ginsenosides Biosynthesis in Ginseng Root by Integration Analysis of Chemical and Transcriptome
Source: Molecules. 2017 May 31;22(6):889. doi: 10.3390/molecules22060889 (PMC6152789; doi:10.3390/molecules22060889)
Supplement: Supplementary file 1 [file molecules-22-00889-s001.zip › molecules-192541-supplementary /Supplementary material 3-Table S2.doc]

**Table S2.** Statistics of transcriptome data for ginseng main root tissues. P, C, and S

refer to the periderm, cortex, and stele, respectively.

| **Tissue** | **Duplicates** | **No. of**  **raw reads** | | **Length (bp)** | **No. of**  **clean reads** | **Length (bp)** | **Q20(%)** | **Q30(%)** |
| --- | --- | --- | --- | --- | --- | --- | --- | --- |
| Periderm | P-1 | 61,695,854 | 9,254,378,100 | | 59,899,416 | 8,889,217,512 | 98.3 | 94.81 |
|  | P-2 | 64,366,314 | 9,654,947,100 | | 62,673,864 | 9,314,763,452 | 98.34 | 94.93 |
|  | P-3 | 66,158,608 | 9,923,791,200 | | 63,976,792 | 9,500,842,960 | 98.23 | 94.66 |
|  | P-4 | 58,068,472 | 8,710,270,800 | | 56,217,766 | 8,349,237,192 | 98.24 | 94.67 |
|  | P-5 | 65,253,120 | 9,787,968,000 | | 63,602,638 | 9,436,696,420 | 98.46 | 95.3 |
| Cortex | C-1 | 84,645,170 | 12,696,775,500 | | 83,206,806 | 12,384,826,951 | 98.76 | 96.06 |
|  | C-2 | 57,060,734 | 8,559,110,100 | | 55,469,298 | 8,241,715,172 | 98.32 | 94.87 |
|  | C-3 | 50,174,138 | 7,526,120,700 | | 48,788,932 | 7,253,421,591 | 98.36 | 94.98 |
|  | C-4 | 62,704,574 | 9,405,686,100 | | 61,079,512 | 9,086,124,072 | 98.45 | 95.25 |
|  | C-5 | 63,561,166 | 9,534,174,900 | | 62,073,612 | 9,234,626,229 | 98.47 | 95.32 |
| Stele | S-1 | 71,410,546 | 10,711,581,900 | | 70,123,394 | 10,432,737,755 | 98.73 | 95.99 |
|  | S-2 | 62,505,778 | 9,375,866,700 | | 60,748,046 | 9,022,519,937 | 98.32 | 94.9 |
|  | S-3 | 65,944,466 | 9,891,669,900 | | 64,250,676 | 9,549,141,452 | 98.37 | 95.02 |
|  | S-4 | 62,604,380 | 9,390,657,000 | | 61,042,960 | 9,085,502,078 | 98.44 | 95.21 |
|  | S-5 | 57,645,514 | 8,646,827,100 | | 56,071,292 | 8,340,008,501 | 98.44 | 95.22 |
| Total | 15 | 953,798,834 | 143,069,825,100 | | 929,225,004 | 138,121,381,274 | - | - |
