# Supplementary material for: Comprehensive Characterization for Ginsenosides Biosynthesis in Ginseng Root by Integration Analysis of Chemical and Transcriptome
Source: Molecules. 2017 May 31;22(6):889. doi: 10.3390/molecules22060889 (PMC6152789; doi:10.3390/molecules22060889)
Supplement: Supplementary file 1 [file molecules-22-00889-s001.zip › molecules-192541-supplementary /Supplementary material 4-Table S3.doc]

**Table S3.** Homology and protein sequence similarity search of the unigene set.

| **Homology search** | **No. of unigenes** |
| --- | --- |
| Nr | 85,928 |
| Swiss-Prot | 78,250 |
| GO | 63,817 |
| KEGG | 49,220 |
| At least one significant match | 90,104 |
