# Supplementary material for: Comprehensive Characterization for Ginsenosides Biosynthesis in Ginseng Root by Integration Analysis of Chemical and Transcriptome
Source: Molecules. 2017 May 31;22(6):889. doi: 10.3390/molecules22060889 (PMC6152789; doi:10.3390/molecules22060889)
Supplement: Supplementary file 1 [file molecules-22-00889-s001.zip › molecules-192541-supplementary /Supplementary material 8-Table S6.doc]

**Table S6.** Statistics of differential expression unigenes between two tissues

(FDR ≤ 0.05 & FC ≥ 2 ).

| **Differential expression unigenes** | **Total** | **Up-regulated** | **Down-regulated** |
| --- | --- | --- | --- |
| Periderm vs Cortex | 3,116 | 2,210 | 906 |
| Periderm vs Stele | 3,311 | 2,357 | 954 |
| Cortex vs Stele | 772 | 457 | 312 |
